# Network analysis of immunotherapy-induced regressing tumours identifies novel synergistic drug combinations

**DOI:** 10.1038/srep12298

**Published:** 2015-07-21

**Authors:** W. Joost Lesterhuis, Catherine Rinaldi, Anya Jones, Esdy N. Rozali, Ian M. Dick, Andrea Khong, Louis Boon, Bruce W. Robinson, Anna K. Nowak, Anthony Bosco, Richard A. Lake

**Affiliations:** 1 National Centre for Asbestos Related Diseases; 2 School of Medicine and Pharmacology, University of Western Australia, The Harry Perkins Institute of Medical Research, 5th Floor, QQ Block, 6 Verdun Street, Nedlands WA 6009, Australia; 3 Telethon Kids Institute, The University of Western Australia, 100 Roberts Road, Subiaco WA 6008, Australia; 4 Bioceros, Yalelaan 46, 3584 CM Utrecht, The Netherlands; 5 Department of Medical Oncology, Sir Charles Gairdner Hospital, Hospital Ave, Nedlands WA 6009, Australia

## Abstract

Cancer immunotherapy has shown impressive results, but most patients do not respond. We hypothesized that the effector response in the tumour could be visualized as a complex network of interacting gene products and that by mapping this network we could predict effective pharmacological interventions. Here, we provide proof of concept for the validity of this approach in a murine mesothelioma model, which displays a dichotomous response to anti-CTLA4 immune checkpoint blockade. Network analysis of gene expression profiling data from responding versus non-responding tumours was employed to identify modules associated with response. Targeting the modules via selective modulation of hub genes or alternatively by using repurposed pharmaceuticals selected on the basis of their expression perturbation signatures dramatically enhanced the efficacy of CTLA4 blockade in this model. Our approach provides a powerful platform to repurpose drugs, and define contextually relevant novel therapeutic targets.

Antibodies blocking immune checkpoint molecules such as cytotoxic T lymphocyte antigen (CTLA) 4 are effective in diverse cancer types, with some patients displaying durable complete regression^1^,^2^,^3^. However, most patients do not show this positive reactivity after treatment. It is not known what molecular events differentiate a response from a lack thereof, nor what treatments might improve response rates^4^. Current strategies are empirical and involve testing different combinations of checkpoint blocking antibodies with other immunotherapeutic strategies such as vaccines or other checkpoint blocking antibodies^5^, or with anti-cancer drugs such as classical chemotherapeutics or oncogenic pathway-targeted small molecular drugs^6^.

We postulated that the dichotomy in response to CTLA4 blockade could be leveraged for increasing treatment efficacy, by visualizing the immunotherapy-induced response in the regressing tumour as a complex, modular network of interacting gene products^7^,^8^. We hypothesized that if we could identify response-associated modules, we could pharmacologically modulate these in order to increase the response rate. Others have used network analysis of gene expression data from tumours to identify drug targets before^9^,^10^, but this has always focussed on the evolving cancer rather than therapy-induced regressing cancer^11^.

Here, we used network analysis of gene expression data from responding versus non-responding tumours from anti-CTLA4 treated mice to identify repurposed drugs that further improve the efficacy of CTLA4 blockade.

## Results

### Mice with bilateral subcutaneous AB1-HA mesothelioma tumours respond symmetrically to anti-CTLA4

**Figure 1:**
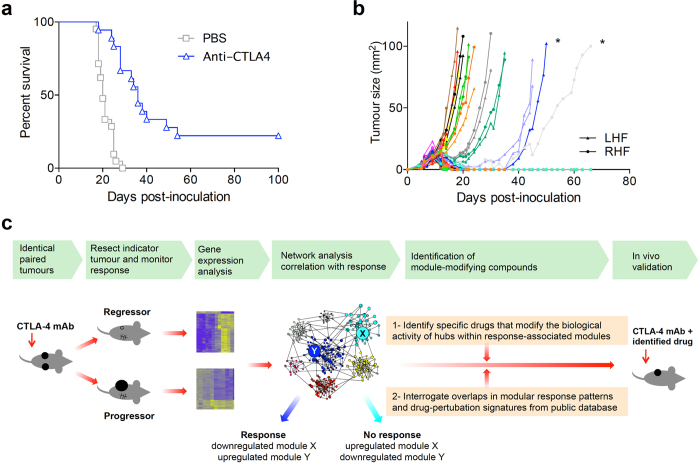
Treatment with CTLA4 blockade results in dichotomous and symmetric responses in identical tumor-bearing mice. (**a**) BALB/c mice were inoculated s.c. with AB1-HA mesothelioma cells on day 0, followed by i.p. injection of 200 μg anti-CTLA4 (n = 18) or PBS on day 6 or 7 (n = 21 mice, pooled data from 2 independent experiments). (**b**) Bilaterally inoculated AB1-HA tumor-bearing mice were treated with anti-CTLA4 on day 5 or 6 (n = 30 mice, pooled data from 2 independent experiments, colour-coded per mouse). Asymmetric responding tumours are marked with an asterisk. (**c**) Graphic representation of the experimental approach.

In studies using anti-CTLA4 to treat mice with subcutaneous AB1-HA mesothelioma tumours^12^, we noticed that some of the mice did not respond while others displayed a rapid regression (Fig. 1a). Although this dichotomous response has been observed by many other groups before, both in mice^13^,^14^,^15^ and in patients treated with anti-CTLA4^1^, this finding struck us as surprising since the mice were genetically identical, experienced the same environment and were treated identically. In single tumour experiments, the outcome for an individual animal is only known at the end of the experiment, by which time the opportunity to study early events underlying regression has been lost. Therefore, we inoculated mice on both flanks with tumours, and observed that the treatment-induced response was symmetrical, in a highly reproducible manner over multiple experiments (Fig. 1b and Supplementary Table 1). Thus, this dual tumour model allowed detailed analysis of the early cellular and molecular events that occur in an anti-CTLA4 responsive tumour, without destroying the outcome readout (the remaining tumour), in the most informative setting: where responses are discordant between identically treated animals.

### Network analysis of responding versus non-responding tumours identifies modules associated with regression

**Figure 2:**
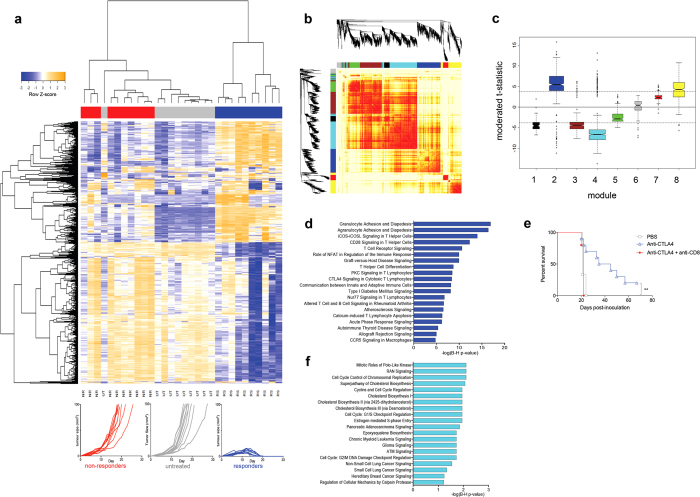
Network analysis of gene expression data from regressing versus non-regressing tumours identifies associated modules. (**a**) Unsupervised hierarchical cluster analysis of microarray data from unilaterally removed AB1-HA tumours from responders (RS), non-responders (NR) and untreated mice (UT), with tumor growth curves. (**b**) A co-expression network was constructed by applying the WGCNA algorithm. Eight modules were identified (tagged by colour coding). (**c**) Modules were related to treatment response by identifying differentially expressed genes between responders and non-responders, and plotting the differential t-statistics as box-and-whisker plots on a module-by-module basis. The dashed horizontal lines correspond to FDR < 0.001. (**d**) Canonical pathways enriched in module 2 (immune module). Data analysis was performed with Ingenuity Systems software. (**e**) AB1-HA bearing mice were treated with anti-CTLA4 200 μg on day 6 after tumor inoculation with or without a CD8 depleting antibody 150 μg i.v., one day before anti-CTLA4, followed by 100 μg i.p. every 3 days, 6 dosages in total (PBS n = 3; anti-CTLA4 n = 10; anti-CTLA4 + anti-CD8 n = 5) (**p < 0.01). (**f**) Canonical pathways enriched in module 4 (cancer module).

We treated mice with anti-CTLA4 or PBS, surgically removed one of the tumours 7 days after treatment administration, at which time regressor and progressor tumours are macroscopically identical. Gene expression profiling by microarray was performed on these tumours and also on PBS treated controls. The data were analysed by unsupervised hierarchical clustering, which revealed that the three experimental groups had distinct gene expression profiles; responders were clustered separate from non-responders and untreated samples (Fig. 2a).

A coexpression network was constructed employing weighted gene co-expression network analysis (WGCNA) (Fig. 2b)^16^,^17^,^18^. This analysis identified 8 modules of highly coexpressed genes operating within the tumours (Fig. 2c and Supplementary Fig. 1). The modules were tested for differential expression in responders versus nonresponders, and this analysis revealed that modules 2 and 4 showed the strongest association with treatment response. Module 2 was upregulated in responders, and this module was enriched for T cell genes, and we therefore designated it the *immune module* (Fig. 2d and Supplementary Table 2). Concordantly, depletion of CD8 T cells completely abrogated the response to anti-CTLA4 (Fig. 2e). Module 4 was downregulated in responders; it consisted principally of genes involved in cell cycle regulation and signalling pathways relevant to cancer development (Fig. 2f and Supplementary Table 3). Accordingly, this module was designated the *cancer module*.

### Pharmacological modulation of response-associated hubs increases response rates to anti-CTLA4

**Figure 3:**
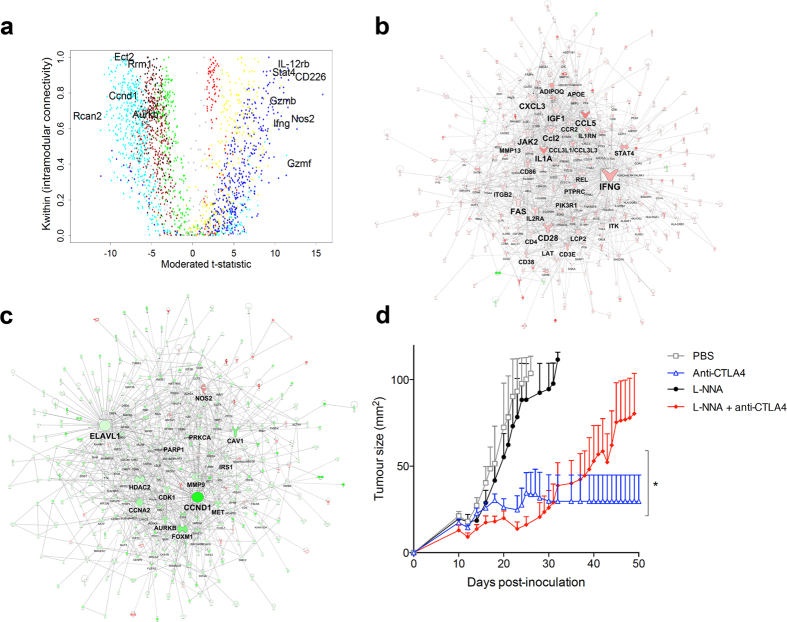
Computational identification of hubs associated with the response to checkpoint blockade. (**a**) Weighted gene correlation network analysis: the x axis depicts the differential expression of the genes, the y-axis the Kwithin value, a measure of connectivity; the genes are colour-coded per module similar to Fig 2c (blue codes for genes within the immune module; cyan for genes within the cancer module) (**b**) Prior knowledge-based graphical reconstruction of the wiring diagram of the immune module and (**c**) the cancer module. (**d**) Tumor growth curve of AB1-HA tumour-bearing mice treated with anti-CTLA4 in combination with competitive NOS2 inhibitor L-NNA, showing abrogation of anti-CTLA4 efficacy.

**Figure 4:**
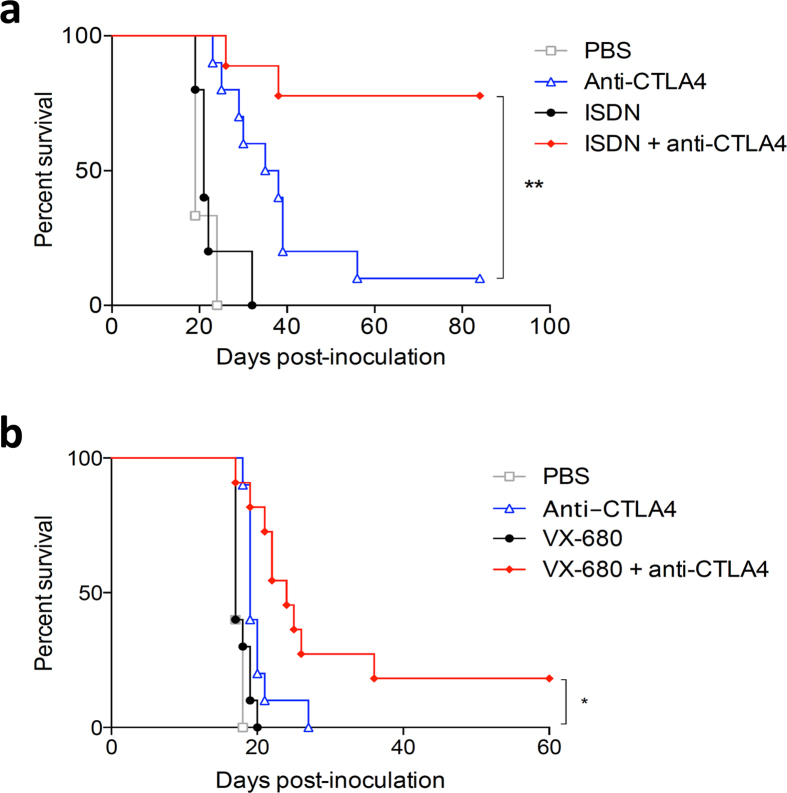
Drugs that target response-associated hubs synergize with anti-CTLA4. (**a**) Co-treatment with ISDN or (**b**) AURKB inhibitor VX680 improved therapeutic efficacy of anti-CTLA4. Representative Kaplan Meier survival curves of 3 independent experiments are shown, n = 10 mice per arm; *p < 0.05; **p < 0.01.

Having identified that there are striking changes in the expression levels of the immune and cancer modules in responders versus non-responders, we reasoned that targeting these modules with drugs could potentially increase the response rate to anti-CTLA-4. We targeted the modules by selectively perturbing the biological activity of hub genes^18^. We used two different approaches to identify hub genes within the response-associated modules. First, we used an unbiased approach, based on overall strength of the correlation patterns between genes within the same module using weighted gene correlation network analysis (Fig. 3a)^18^. In the second approach, we employed experimentally supported molecular interaction data from prior studies to reconstruct the wiring diagram of the modules, using the Ingenuity Systems Knowledgebase^19^. This analysis identified major hub genes in the immune and cancer modules (Fig. 3b,c). Nitric oxide synthase 2 (NOS2) was one of the highest ranked hubs in both independent analyses. Although our analysis identified NOS2 as a hub in the cancer module, it was highly upregulated in responders, while the majority of genes in that module were downregulated, suggesting a reciprocal relationship with other genes in the module (Fig. 3a,b). NOS2 is a transcriptionally regulated isoform of NOS, which catalyses the production of NO from L-arginine, particularly in response to cytokines, generating sustained, high output quantities of NO^20^. To determine if NOS2 plays a major role in the response to CTLA-4 blockade, we treated mice with established AB1-HA tumours with anti-CTLA-4 in combination with competitive NOS2 inhibitor L-N^G^-nitroarginine. The data show that treatment efficacy decreased significantly when this hub was inhibited (Fig. 3d). Having established a role for NOS2 in treatment response, we then wanted to determine if this pathway could be harnessed to increase the response rate. Given that selective drugs that enhance NOS2 activity are not available, we used isosorbide dinitrate (ISDN) as a NO generator. ISDN treatment alone did not have any effect on tumour outgrowth, but in combination with anti-CTLA-4 it very significantly improved the cure rate from 10% to 80%, displaying a clear synergistic effect (Fig. 4a).

We then searched for other hubs to target in the cancer module, for which selective drugs are readily available. We identified Aurora Kinase B (AURKB) as a plausible target in this regard. It was identified as a hub of the cancer module, and was downregulated in responders. Indeed, we found that downregulation of AURKB using the specific inhibitor VX680 significantly increased treatment efficacy over anti-CTLA-4 alone (Fig. 4b).

These findings suggest that pharmacological modulation of hubs identified within treatment response-associated modules can improve the response rate to the initial treatment.

### Repurposed drugs that are known to modulate expression levels of genes in response-associated modules can increase response rates to anti-CTLA4

**Figure 5:**
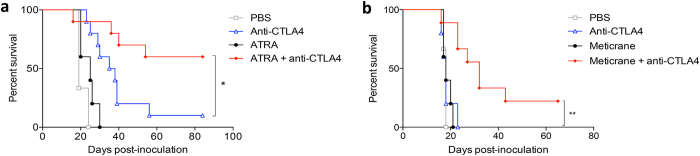
Repurposed drugs that phenocopy the gene expression profile of response-associated modules synergize with anti-CTLA4. Survival curve of AB1-HA tumour-bearing mice treated with anti-CTLA4 in combination with (**a**) ATRA and (**b**) meticrane improved therapeutic efficacy of anti-CTLA4. Representative survival curves of 3 independent experiments are shown, n = 10 mice per arm; *p < 0.05; **p < 0.01.

Given that selective drugs are often not available to target hub genes, we wanted to explore an alternative and more generalized approach to find drugs that can increase therapeutic efficacy. To address this issue, we hypothesized that repurposed drugs that modulate expression levels of multiple genes within the response modules may also improve treatment response. Two different computational tools were employed for this analysis. The first approach utilized the connectivity map (cMap) database^21^,^22^, which contains genome-wide expression signatures of cell lines induced by 1309 compounds^22^ (Supplementary Table 4). The second approach utilized upstream regulator analysis^23^, which identifies drugs that are known *a priori* to modulate the expression levels of genes that are differentially expressed in a data set, and additionally measures the pattern match between the observed expression changes (up/down regulation) and the predicted pattern from prior knowledge (Supplementary Table 5). In both analyses retinoic acid was identified as a potential candidate. Retinoic acid exerts differential effects on immune cells, including effector T cells, macrophages, dendritic cells and suppressive cell subsets^24^,^25^,^26^. All-*trans* retinoic acid (ATRA) is used in acne skin disease and in promyelocytic leukemia as stem cell differentiation inducer, but it has never been used in combination with checkpoint blockade before. *In vivo* validation experiments showed a significant enhancement of treatment efficacy when ATRA was co-administered with CTLA-4 blockade, with long-term cures in the majority of mice (Fig. 5a). In addition, we tested multiple other drugs based on the ranking within the cMap analysis and availability. Meticrane, a thiazide diuretic was highly ranked in the cMap analysis, and it does not have any known anti-cancer or immune-stimulating effect. Co-treatment with meticrane significantly enhanced treatment efficacy of CTLA-4 blockade (Fig. 5b).

Overall, using both hub-targeted and module-targeted repurposed drugs or biologicals, 4 out of 14 tested drugs showed a clear and significant improvement over anti-CTLA4 alone (Figs 4 and 5), while a further 3 displayed a consistent trend of additive anti-tumour effect (Supplementary Fig. 2).

These data demonstrate that drugs which are known to modulate expression levels of genes in the response-associated modules in other experimental settings can increase response rates to CTLA4 blockade.

## Discussion

The molecular mechanisms that determine an effective anti-tumour response after immune checkpoint blockade are poorly understood. It is therefore not possible to rationally target these mechanisms to further improve on the efficacy of current immune checkpoint blocking treatments. Here, we developed an experimental strategy to characterize the molecular networks that are operating within the tumor to mediate anti-CTLA4 induced tumor regression, and identify drugs that target these mechanisms and are able to increase therapeutic response rates.

There are several novel aspects to our studies that are noteworthy. Firstly, while others have used network analysis of gene expression data before to elucidate the molecular processes that govern the invasive and proliferative potential of cancers while they evolve^9^, we instead focussed on cancers while they were regressing^11^.

Second, we harnessed and exploited the dichotomous nature of the response to checkpoint blockade that has been observed by many different research groups using multiple different mouse tumour models to identify the response mechanisms^13^,^14^,^15^. The observation that a module of immune related genes was upregulated in responders, while a module consisting of genes associated with cancer was downregulated is perhaps not surprising. However, subsequent detailed mapping of these modules allowed identification and validation of drugs that were not obvious candidates in this context at the outset of the study.

Third, the dual cancer model with symmetrically responding bilateral tumours allowed us to identify potential mediators of response in tumours and mice that were identical in every known aspect, except for their response to therapy. By combining this model with a network analysis approach, we could identify and prioritize several hubs that were associated with response. The hubs that we thus identified are likely context dependent and included genes that were not previously associated with a response to checkpoint blockade. For example, some studies found a tumour-promoting role for NO^27^ and in others, NO inhibitors have been proposed as chemopreventive agents^28^. Our data suggest that in the context of immunotherapeutic regression, the amplification of the biological activity of NO can in fact be beneficial. We internally validated the relevance of these hubs, within the same tumour model, that modification of these hubs indeed changed the response rate to anti-CTLA4.

Fourth, we applied a drug repurposing approach to the response-associated network data. This resulted in the identification of pleiotropic drugs that improved the response rate to the backbone therapy, anti-CTLA4. One of the repurposed drugs that we identified in this manner, meticrane, has not been associated with any anti-tumour or pro-immune effect and could not have been predicted to work in synergy with anti-CTLA4 otherwise. There lies tremendous potential in the use of repurposed drugs: these drugs have been shown to be safe and are routinely used by millions of people worldwide, they often are cheap and readily available and could thus be rapidly translated into clinical trials, which all together results in a substantial shortening of the drug developmental trajectory^29^. We note that our gene expression data were obtained *in vivo* from mesothelioma-bearing mice, while the cMap dataset was obtained using human cell lines from non-mesothelioma cancers cultured *in vitro*. We also note that even though we did not extensively optimize the dosing and scheduling of the repurposed compounds, 7 out of 14 tested drugs that we predicted to work in concert with anti-CTLA4, indeed showed an additional anti-tumour effect. This is a very high ‘hit rate’ going from *in silico* identification to biological *in vivo* effect, in the absence of severe toxicity^30^.

Our study has limitations that should be acknowledged. We focused on a single time point in a mixed cell population, using a single mouse tumour model. Future studies will be required to further dissect the role of different cell populations and their dynamic interactions. It also remains to be established whether the results can be directly translated to different cancers and to cancer patients, or whether the network analysis studies should be carried out on patient samples during therapy to identify the most relevant and potent drugs.

Notwithstanding these limitations, our approach appears especially attractive for drug discovery since our analysis captures mechanisms operating in the tumour in its entirety, whilst pinpointing therapeutic targets, which can be selectively modulated to modify response. In addition, the technology could be used as a starting point for biological investigations into the mechanism of action of drugs, for example into the role of iNOS in anti-CTLA4 induced immunity. We postulate that this methodology can potentially be used in other diseases that display a dichotomous response to treatment.

## Methods

### Mice

BALB/c (H-2d) mice were obtained from the Animal Resources Centre (Murdoch, Western Australia) and were maintained under specific pathogen free (SPF) conditions (M-Block Animal Facility, Queen Elizabeth II Medical Centre, The University of Western Australia). All mice used in these studies were female and between 8–12 weeks of age. All animal experiments were conducted according to The University of Western Australia Animal Ethics Committee approvals (protocol RA/3/100/1139) and the code of conduct of the National Health and Medical Research Council of Australia.

### Cell Lines

The MHC class I-positive, class II-negative, highly tumorigenic and poorly immunogenic BALB/c-derived asbestos-induced mouse mesothelioma cell line AB1, transfected with the influenza HA gene (AB1-HA) has been described before^12^,^31^,^32^. The cell line was validated by CellBank Australia. Cell lines were maintained in RPMI 1640 (Invitrogen, Mulgrave, Australia) supplemented with 20 mM HEPES, 0.05 mM 2-mercaptoethanol, 100 units/ml penicillin (CSL, Melbourne, Australia), 50 μg ml^−1^ gentamicin (David Bull Labs, Kewdale, Australia), and 10% FCS (Invitrogen). AB1-HA cells were maintained in media containing the neomycin analogue geneticin (Invitrogen) at a final concentration of 400 μg ml^−1^. All cell lines were regularly tested and remained negative for Mycoplasma spp.

### Antibodies

The anti-CTLA4 (clone 9H10) monoclonal antibody was prepared and purified at the Monoclonal Antibody Facility, WAIMR (Perth, Australia) and by Bioceros (Utrecht, The Netherlands). The anti-CTLA4 hybridoma was a kind gift from Prof. J.P. Allison (Memorial Sloan Kettering Cancer Centre, New York, US). Anti-CD8 (clone YTS169.4) monoclonal antibody was prepared and purified at the Monoclonal Antibody Facility, WAIMR (Perth, Australia).

### Tumor challenge and anti-CTLA4 treatment protocols including surgery

Initial experiments into symmetry of response: BALB/c mice were inoculated subcutaneously (s.c.) with 5 × 10^5^ AB1-HA cells on both flanks on day 0. They were treated with anti-CTLA4 on day 5 or 6 intraperitoneally (i.p.). In these experiments we aimed for a response rate of approximately 40–60%, but depending on the batch of the anti-CTLA4 antibody we observed slight differences in response rates. We therefore optimized dosing for each subsequent batch, using either single dose of 100 or 200 μg per mouse. Non-responders and responders were matched for dose (Table S1). Using this approach, responses were highly symmetric: mice either displayed complete tumor regression on both sides, or no response on either side. In only 13% of the mice the response was asymmetric: regrowth appeared in a previously responding one-sided lesion, which always occurred within 40 days (Fig. 1b and Table S1). In responding animals, tumours consistently started regressing on day 7 after administration of anti-CTLA4; before this time point tumours of responding and non-responding mice were identical in size (Fig. 1b). Sham surgery did not affect the symmetry of response (data not shown). Therefore, we concluded that we could remove one tumor, leaving the other *in situ* and subsequently infer the fate of the tumor we have removed by monitoring the growth of the other tumor for at least 40 days.

Subsequent experiments with unilateral tumor removal: We chose to remove tumours from one side of bilaterally inoculated animals 7 days after anti-CTLA4 administration, because at this time there was no difference in size between those destined to respond to therapy and those that would not. Tumors from 4 different experiments were used, in which the mice were treated on day 5 or 6 with 200 μg anti-CTLA4. Mice were anesthetized with isofluorane (2% in oxygen) for not more than 10 minutes, during which one s.c. tumor was removed through an incision in the flank and immediately fully submerged in RNAlater (Life Technologies, Australia). The surgical wounds were sutured using 5/0 vicryl continuous sutures (Ethicon, North Ryde, Australia). Mice were placed under a heat lamp for recovery.

Over the following weeks the remaining indicator tumor was measured using digital micro-calipers at least three times per week and based on its growth characteristics the mice were divided into three different groups:

#### 1-Responders

The indicator tumor was evident as a palpable subcutaneous nodule of at least 5 mm^2^ on the day of surgery of the other tumor (which also should be palpable) and it regressed to 0 mm^2^ and stayed undetectable for more than 2 months post-inoculation.

#### 2-Non-responders

The indicator tumor was evident as a palpable subcutaneous nodule of at least 5 mm^2^ on the day of surgery of the other tumor (which also should be palpable) and it continued to progress, i.e. there was no sign of slowing of growth or a partial decrease. When the tumor size reached 100 mm^2^ mice were euthanized following regional animal ethics guidelines.

#### 3-Partial responders

Not a clear responder or non-responder, i.e. there was either a full regression of the indicator tumor but this was followed by tumor outgrowth within the observed time period (of at least 2 months), partial regression, delayed or slowed outgrowth or a tumor of <5 mm^2^ at time of surgery. These mice were excluded from subsequent analyses.

As a control group, tumours were removed 7 days after sham treatment with 100 μl PBS on day 6 after tumor inoculation.

We performed gene expression microarray analysis comparing anti-CTLA4-treated mice that had shown full regression of the contralateral tumor without reccurrence in the following 2 months (responders, n = 10) with mice that had continuous growth of the contralateral tumours (non-responders, n = 10). We used PBS-treated mice as control (n = 10).

### RNA isolation

The tumours were stabilized in RNA*Later* (Life Technologies, Australia) and stored at −80 °C. The tumours were dispersed in TRIzol (Life Technologies, Australia) employing a TissueRuptor rotor-stator homogenizer (QIAgen, Australia). After addition of chloroform and aqueous phase separation, the samples were purified on RNeasy MinElute columns (QIAgen, Australia). The integrity of the RNA samples was confirmed on the Bioanalzyer (Agilent Technologies, USA).

### Microarrays

Total RNA samples from the 3 experimental groups of mice (responders, non-responders, PBS controls) comprising 10 mice/group (total sample size = 30) were labeled and hybridized to Mouse Gene 1.0 ST microarrays (Affymetrix, USA) at the Ramaciotti Centre for Gene Function Analysis (University of New South Wales, Australia). The microarray data was high quality; mean raw intensity of pm probes (±SD) = 398.2 ± 89.8; discrimination of positive versus negative control probes = 0.85 ± 0.02; median absolute deviation of the residuals mean = 0.33 ± 0.05; relative log expression mean = 0.23 ± 0.07. The raw microarray data are available from the Gene Expression Omnibus data repository (accession: GSE63557).

### Network analysis

The microarray data was preprocessed in R employing the Factor Analysis for Robust Microarray Summarization (qFARMS) algorithm^33^. A custom chip description file (mogene10stmmentrezg, version 18) was used to map probe sets to genes based on current genome annotations^34^. The informative/non-informative calls algorithm was employed to filter out noisy probe sets^35^. A coexpression network was constructed employing the weighted gene coexpression network analysis algorithm (WGCNA)^18^,^36^. Genes/modules associated with response to treatment were identified using moderated *t*-statistics, with False Discovery Rate control for multiple testing^37^. The wiring diagram of the modules was reconstructed using the Ingenuity Systems KnowledgeBase of expert curated functional data from published studies (Ingenuity Systems Knowledgebase)^19^. Hubs were prioritized for drug targeting studies by counting the number of genes they were connected to (degree) in the wiring diagram. Hubs were also prioritized by plotting the gene expression data along axes of differential expression and intramodular coexpression network connectivity^18^,^36^.

### Drug repositioning using cMap database

This analysis was based on the connectivity map (cMap) database, which comprises gene expression profiles from a panel of human cell lines induced by 1,309 drug compounds^22^. Modules associated with response to treatment were identified as described above. Human orthologs of the mouse genes within these response modules were identified using a conversion table from the Mouse Genome Informatics database. Human orthologs were then mapped to Affymetrix hgu133a probe sets using annotation packages from Bioconductor. Up and down regulated probe sets were defined by contrasting gene expression levels in tumours from responder versus nonresponder mice. The probe sets were queried against the cMap build 02 database using software from the Broad Institute (https://www.broadinstitute.org/cmap/).

### Drug repositioning using Upstream Regulator Analysis

The genes in the immune/cyan modules and their log2 fold change values were analyzed using the Ingenuity Systems Upstream regulator analysis algorithm^23^. The algorithm identifies drugs that based on prior experimental evidence are capable of driving (or reversing) the observed gene expression patterns detected in an experiment. An overlap p-value is calculated based on enrichment of known target genes for each given drug that are differentially expressed in the experiment. An activation Z-score is also calculated which measures the pattern match between the direction of the gene expression changes (up-down regulation) and the predicted pattern based on prior evidence.

### Drugs and treatment schedules for *in vivo* treatment

For *in vivo* intervention studies using network-targeted agents BALB/c mice were inoculated with 5 × 10^5^ AB1-HA cells s.c. on one flank. Anti-CTLA4 was administered i.p. on day 10 at a single dose of 100 μg per mouse. The studies were optimized to give a response rate of around 10% for anti-CTLA4 alone. Between experiments this varied between 0–20%. The following drugs were administered in combination with anti-CTLA4. All drugs were started with dosing on day 10, together with the anti-CTLA4 unless otherwise indicated. The dosages were based on published studies, unless otherwise stated. Tumors were measured using digital micro-calipers at least three times per week. When the tumor size reached 100 mm^2^ mice were euthanized following regional animal ethics guidelines.

*Anti-CD8* was administered as described previously^12^. Briefly, 150 μg of anti-CD8 was given i.v., one day before anti-CTLA4, followed by 100 μg i.p. every 3 days for 6 dosages (last dose on day 20).

*L-NNA* (Cayman Chemicals, Mic, USA) was dissolved in PBS to a concentration of 1 mg ml^−1^ and sonicated to dissolve. Mice received i.p. injections of 15 mg kg^−1^ bodyweight every second day for 10 doses, post anti-CTLA4 treatment. This dose regime was based on literature^38^, combined with our own dose-optimizing study in which we sequentially treated 2 groups (n = 3 per group) of standard BALB/c mice with 15 mg kg^−1^ or 30 mg kg^−1^ for 10 days and monitored weight and clinical scores. We observed weight loss of around 15% with the highest dose (data not shown) and therefore decided to use the lower dose.

*Isosorbide dinitrate* (Toronto Research Chemicals Inc, To, Canada) was dissolved to 4 mg ml^−1^ in DMSO. Mice received daily i.p. injection for 14 days at 200 μg per mouse post anti-CTLA4 treatment^39^. Isosorbide dinitrate is commonly used in coronary artery disease and congestive heart failure.

*All-trans-retinoid acid* (Selleck Chemicals, Tx, USA) was dissolved in DMSO to a concentration of 100 mg ml^−1^ in DMSO and diluted to a final concentration of 4 mg ml^−1^ in PBS. Mice received daily i.p. injections at a dose of 10 mg/kg bodyweight for 10 days post anti-CTLA4 treatment^40^. ATRA is commonly used in acne skin disease and in promyelocytic leukemia as stem cell differentiation inducer.

*VX-680* (AdooQ Bioscience, CA, USA) was dissolved in DMSO to a concentration of 32 mg ml^−1^. Mice received daily i.p. injections at a dose of 80 mg/kg bodyweight for 14 days post anti-CTLA4 treatment^41^. We observed substantial toxicity when we treated mice simultaneously with VX-680 and anti-CTLA4 (sterile inflammation of the peritoneal cavity leading to bowel obstruction as found by macroscopic and microscopic examination; there was no sign of colitis, data not shown). For this reason, we decided to repeat the experiments in a sequential manner with the VX-680 given daily for 10 days, starting 5 days after the anti-CTLA4.

*Meticrane* (Sigma, MO, USA) was dissolved in DMSO to a concentration of 160 mg ml^−1^. Mice received daily i.p. injections at a dose of 400 mg/kg bodyweight for 10 days post anti-CTLA4 treatment. This dose was based on our own dose-optimizing studies, since we could not find dosing studies in mice in the literature. The thiazide diuretic meticrane is approved as an antihypertensive in Japan and is used at doses of 150–300 mg once daily; the reported LD50 for mice is 10 g kg^−1^ after i.p. administration^42^,^43^. We treated 3 groups of standard BALB/c mice consecutively (n = 3/group) with increasing doses of meticrane i.p. (100 mg kg^−1^; 200 mg kg^−1^ and 400 mg kg^−1^) for 10 days and monitored weight and general wellbeing. The dose of 400 mg kg^−1^ was the highest dose achievable before observing slight weight loss (data not shown), but still in the absence of other clinical signs of toxicity.

*Hydrocotamine* (Indofine Chemical Company, NJ, USA) was dissolved in PBS to a concentration of 80 g/ml and mice received daily i.p. injections at a dose of 0.4 mg kg^−1^ bodyweight for 14 days post anti-CTLA4 treatment^44^.

*Fasudil* (Selleck Chemicals, Tx, USA) was dissolved to 2 mg/ml in distilled H2O and placed in drinking water of mouse cage from day of anti-CTLA4 treatment, for 14 days^45^.

*Galantamine* (Abcam, Tx, USA) was dissolved to a concentration of 1 mg/ml in PBS. Mice received daily i.p. injections at a dose of 5 mg kg^−1^ bodyweight for 10 days post anti-CTLA4 treatment^46^.

*Pyridoxine* (Abcam, Tx, USA) was dissolved to a concentration of 20 mg ml^−1^ in PBS. Mice received daily i.p. injections at a dose of 100 mg kg^−1^ bodyweight for 7 days post anti-CTLA4 treatment^47^.

*Flavopiridol* (Cayman Chemicals, Mic, USA) was dissolved in DMSO to a concentration of 3 mg ml^−1^. Mice received daily i.p. injections at a dose of 7.5 mg kg^−1^ bodyweight for 10 days post anti-CTLA4 treatment^48^.

*Tomatidine* (Sigma, MO, USA) was dissolved in PBS to a concentration of 1 mg/ml. Mice received daily dosing of 10 mg kg^−1^ bodyweight by oral gavage for 10 days post anti-CTLA4 treatment^49^.

During treatment mice were closely monitored and culled if significant weight loss (≥20% from starting weight) or other significant toxicity was observed. Mice that were tumor-free for more than 3 months after treatment were re-challenged with 5 × 10^5^ AB1 mesothelioma cells that did not express the HA antigen. All animals rejected this second challenge (data not shown), indicating that the anti-CTLA4/drug-induced rejection of the AB1-HA tumor was not dominated by a T cell response towards the HA neo-antigen, which is in line with previously published findings^50^.

### Recombinant cytokines for *in vivo* treatment

Recombinant murine IL-12 (Peprotech, NJ, USA), IL-18 (Novus Biologicals, CO, USA), IL-1β (Novus Biologicals, CO, USA) and IFNγ (Peprotech, NJ, USA) were diluted in PBS and injected i.p. in the following dosages/schedules: IL-12, 0.2 μg for 10 days post anti-CTLA4 treatment; IL-18 0.4 μg for 10 days post anti-CTLA4 treatment; IL-1β 0.5 μg for 10 days post anti-CTLA4 treatment and IFNγ 2 μg for 10 days post anti-CTLA4 treatment.

### Statistical Analyses of Mouse Experiments

The power calculation for all experiments was based on preliminary experiments, allowing the detection of a difference in response rates of 10% compared to 50% in control and experimental groups. We estimated that group sizes of 19 mice would be required to detect this difference with a probability (power) of 0.8 at an alpha of 0.05. For logistical reasons, and in order to assess reproducibility, this was done in two separate experiments of 10 mice per group.

Tumor growth data were analyzed using the PASW statistics version 18 MIXED procedure (IBM SPSS, Chicago IL). Comparisons between treatment groups at each time point were adjusted for multiple comparisons by the Sidak method. Tumor growth curves are depicted in time until all mice had shown either full regression or had reached maximum allowed tumor size.

Survival data were analyzed using Prism 4.0 (GraphPad Software, Inc.), according to the Kaplan Meier method and survival proportions were compared between groups using a Log Rank Test.

## Additional Information

**Accession codes:** The raw microarray data are available from the Gene Expression Omnibus data repository (accession: GSE63557). 

**How to cite this article**: Lesterhuis, W. J. *et al.* Network analysis of immunotherapy-induced regressing tumours identifies novel synergistic drug combinations. *Sci. Rep.* **5**, 12298; doi: 10.1038/srep12298 (2015).

## Supplementary Material

Supplementary Information
