# Supplementary material for: Network analysis of immunotherapy-induced regressing tumours identifies novel synergistic drug combinations
Source: Sci Rep. 2015 Jul 21;5:12298. doi: 10.1038/srep12298 (PMC4508665; doi:10.1038/srep12298)
Supplement: Supplementary Information [file srep12298-s1.pdf]

Supplementary Materials

Network analysis of immunotherapy-induced regressing tumours identifies novel synergistic drug combinations

W. Joost Lesterhuis[1,2]*, Catherine Rinaldi[1,2], Anya Jones[3], Esdy N. Rozali[1,2], Ian M. Dick[1,2], Andrea Khong[1,2], Louis Boon[4], Bruce W. Robinson[1,2], Anna K. Nowak[2,5], Anthony Bosco[3‡], Richard A. Lake[1,2‡]

Affiliations:

[1]National Centre for Asbestos Related Diseases and [2]School of Medicine and Pharmacology, University of Western Australia, Australia

[3]Telethon Kids Institute, The University of Western Australia, Australia

[4]Bioceros, The Netherlands

[5]Department of Medical Oncology, Sir Charles Gairdner Hospital, Australia.

Response	Number of mice (total n=80)	Percentage
Complete regression on both sides (Responders)	36	45%
Did not respond on any side (Non-Responders)	33	41.25%
Responded on one side (Mixed Responders)	11	13.75%

Supplementary Table 1. Symmetry of response of bilaterally inoculated AB1-HA tumours. Mice were injected with 1 x 10^6 AB1-HA cells on day 0 and treated with anti-CTLA4 100 - 200 μg on day 5 or 6. Pooled results from 5 independent experiments are shown.

Note that the mice that displayed a progressing lesion on one side, always did so before day 40 after tumour inoculation (Fig. 1b in article); by observing tumour growth of the indicator tumour for longer than that period, we could thus further decrease the possibility of resecting a non-symmetric responding tumour.

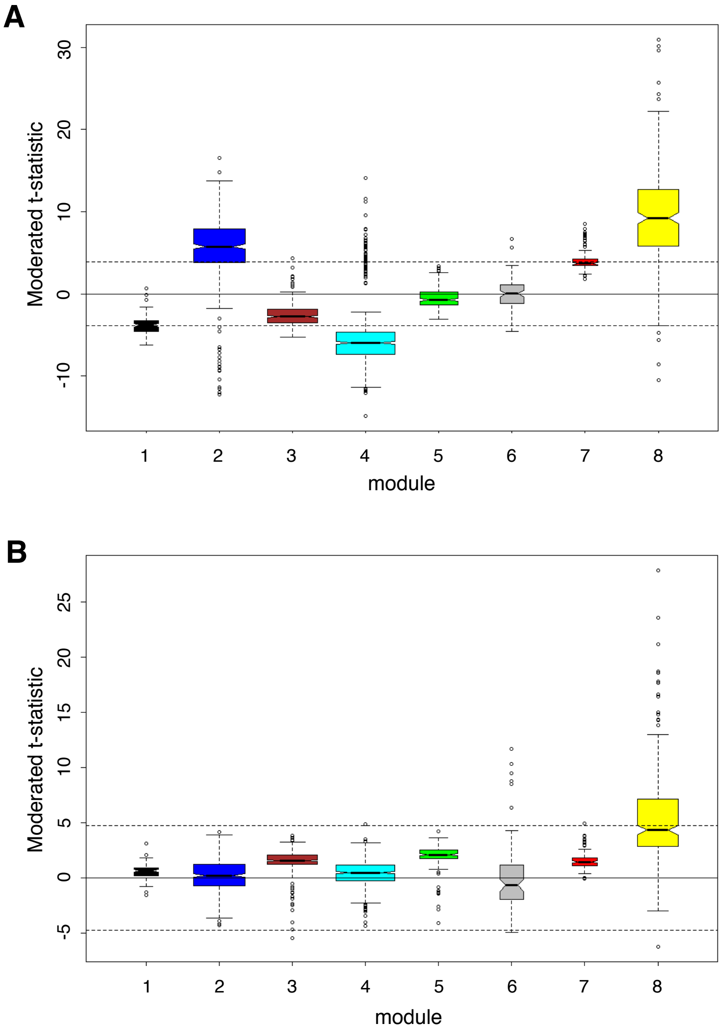

Supplementary Fig. 1. Differential expression of the modules in (**a**) responders versus untreated and (**b**) non-responders versus untreated. Although module 8 was differentially expressed comparing responders with non-responders (Fig. 2c in article), it was also strongly upregulated in tumors from nonresponders compared to PBS controls (in **c**). Modules 2 and 4 were not differentially expressed when comparing non-responders with untreated (in **b**), while it was significantly different between responders and untreated (in **a**) and responders and non-responders (Fig. 2c in article). Therefore, modules 2 and 4 were most differentially expressed between responders and non-responders.

	Genes	#list	#ref_list	adjusted pValue	Silhouette	Terms
Metagroup 1	C3 Camk4 Cblb Ccl2 Ccl22 Ccl3 Ccl5 Ccl6 Ccl8 Ccl9 Ccr2 Ccr3 Ccrl2 Cd24a Cd38 Cd4 Cd52 Cd55 Cd74 Clec4e Clec7a Ctla4 Cxcl11 Cxcl12 Cxcl16 Cxcl2 Cxcl3 Cxcr2 Cxcr3 Dock2 Ear11 Ear2 Enpp2 Fas Fcer1g Fcgr2b Fcgr3 Fpr1 Fpr2 H2-Aa H2-Ab1 Ifng Il1a Itga1 Itgb2 Jak2 Lat Lipa Ly86 Ly96 Nckap1l P2ry14 Pf4 Pik3cg Pla2g7 Prkcq S100a9	57(396)	428(37681)	4.54E-45	0.2694	GO:0006955 immune response (BP)<br>GO:0006935 chemotaxis (BP)<br>GO:0006954 inflammatory response (BP)<br>GO:0030593 neutrophil chemotaxis (BP)<br>GO:0007204 elevation of cytosolic calcium ion concentration (BP)<br>GO:0030595 leukocyte chemotaxis (BP)
Metagroup 2	Abca1 Acsl1 Apoe Arhgap15 B3galt2 Ccl2 Ccr2 Cd226 Cd24a Cd36 Cd4 Cxcl12 Dgat2 Egr2 Enpp2 Fabp4 Gm2a Gnao1 Gpc3 Gpr171 Gpr174 Hsd11b1 Igf1 Il33 Iqgap2 Itk Jak2 Klrk1 Lipa Lipn Lpl Mmp3 Nexn Nr1d1 P2ry10 P2ry14 Pecam1 Pla2g7 Plbd1 Plin2 Psap Ptprc Rel Rgs1 Rgs2 Rpn1 St8sia4 St8sia6 Stat4 Timp3 Tnfaip3	51(396)	746(37681)	2.92E-26	-0.0443	GO:0001816 cytokine production (BP)<br>GO:0071222 cellular response to lipopolysaccharide (BP)<br>GO:0030334 regulation of cell migration (BP)<br>GO:0019915 lipid storage (BP)<br>GO:0007186 G-protein coupled receptor protein signaling pathway (BP)<br>GO:0035589<br>GO:0042632 cholesterol homeostasis (BP)<br>GO:0050729 positive regulation of inflammatory response (BP)<br>GO:0071310 cellular response to organic substance (BP)<br>GO:0016042 lipid catabolic process (BP)<br>GO:0006486 protein amino acid glycosylation (BP)<br>GO:0043410 positive regulation of MAPKKK cascade (BP)<br>GO:0006631 fatty acid metabolic process (BP)<br>GO:0030324 lung development (BP)<br>GO:0043547 positive regulation of GTPase activity (BP)
Metagroup 3	Adipoq Cblb Ccl3 Cd2 Cd24a Cd28 Cd36 Cd3d Cd3e Cd3g Cd4 Cd48 Cd74 Cd86 Cd8a Cd97 Cxcr2 Dock2 Dpp4 Efna5 Emr4 Fcer1g Fcgr2b Fcgr3 Igf1 Jak2 Klrg1 Osmr Satb1 Trat1	30(396)	221(37681)	2.46E-24	0.0612	GO:0042110 T cell activation (BP)<br>GO:0007166 cell surface receptor linked signaling pathway (BP)<br>GO:0050731 positive regulation of peptidyl-tyrosine phosphorylation (BP)<br>GO:0050850 positive regulation of calcium-mediated signaling (BP)
Metagroup 4	Ccl2 Ccl5 Ccr2 Cd24a Cd28 Cd3e Cd4 Cd86 Clec2i Fas Ifng Il12rb2 Il1a Il2ra Itk Klrk1 Malt1 Nckap1l Pdcd1lg2 Prkcq Ptprc Skap1 Themis	23(396)	126(37681)	5.71E-22	0.1438	GO:0042102 positive regulation of T cell proliferation (BP)<br>GO:0050870 positive regulation of T cell activation (BP)<br>GO:0050852 T cell receptor signaling pathway (BP)<br>GO:0046641 positive regulation of alpha-beta T cell proliferation (BP)<br>GO:0045086 positive regulation of interleukin-2 biosynthetic process (BP)<br>GO:0008624 induction of apoptosis by extracellular signals (BP)<br>GO:0032729 positive regulation of interferon-gamma production (BP)
Metagroup 5	Ccl2 Ccr2 Ccr3 Cd74 Csf2rb Cxcr2 Fpr1 Fpr2 Il12rb2 Il1a Il2ra Il2rb Il7r Irak3 Jak2 Lifr Osmr Pf4 Prlr Stat4	20(396)	112(37681)	4.51E-19	0.2054	GO:0019221 cytokine-mediated signaling pathway (BP)<br>GO:0007186 G-protein coupled receptor protein signaling pathway (BP)<br>GO:0006935 chemotaxis (BP)
Metagroup 6	Adipoq Aif1 Camk4 Ccl3 Ccl5 Ccr2 Ces1d Ctsh Cxcl12 Cxcl16 Cyp1b1 Fas H2-Aa Il12rb2 Il33 Il7r Jak2 Kynu Lifr Myo1f Nfil3 Osmr Pf4 Pik3cg Pik3r1 Pla2g7 Prkcq Serpina3g Serpina3n Slc7a11 Sp100	31(396)	381(37681)	1.82E-18	0.1721	GO:0034097 response to cytokine stimulus (BP)<br>GO:0090026 positive regulation of monocyte chemotaxis (BP)<br>GO:0030335 positive regulation of cell migration (BP)<br>GO:0034341 response to interferon-gamma (BP)<br>GO:0006954 inflammatory response (BP)<br>GO:0006955 immune response (BP)<br>GO:0010628 positive regulation of gene expression (BP)<br>GO:0009636 response to toxin (BP)<br>GO:0006935 chemotaxis (BP)<br>GO:0034612 response to tumor necrosis factor (BP)
Metagroup 7	Aif1 Ccl5 Cd36 Cd86 Cd8a Clec5a Clec7a Creg1 Cxcl12 Cxcl9 Fcer1g Gpc3 Ifitm1 Ifng Irak3 Itgax Itgb2 Jak2 Klrk1 Lyz1 Lyz2 Myo1f Pf4 Pglyrp1 Ptprc Ptx3 Rnasel Samhd1 Socs2 Sod2	30(396)	407(37681)	9.98E-17	0.0396	GO:0051607 defense response to virus (BP)<br>GO:0045429 positive regulation of nitric oxide biosynthetic process (BP)<br>GO:0032760 positive regulation of tumor necrosis factor production (BP)<br>GO:0050830 defense response to Gram-positive bacterium (BP)<br>GO:0040008 regulation of growth (BP)<br>GO:0042742 defense response to bacterium (BP)<br>GO:0045666 positive regulation of neuron differentiation (BP)<br>GO:0009615 response to virus (BP)
Metagroup 8	Cd274 Cd74 Ctla4 Ctse Fcer1g Fcgr2b H2-Aa H2-Ab1 H2-Eb1 Ifi30 Ifng Il2ra Pdcd1lg2 Ptprc	14(396)	60(37681)	1.78E-15	0.3496	GO:0019886 antigen processing and presentation of exogenous peptide antigen via MHC class II (BP)<br>GO:0006955 immune response (BP)<br>GO:0042130 negative regulation of T cell proliferation (BP)<br>GO:0045582 positive regulation of T cell differentiation (BP)<br>GO:0019882 antigen processing and presentation (BP)
Metagroup 9	C1s C3 Cd55 Cfb Clec4d Clec4n Clec5a Clec7a Cst7 Cybb Gpc3 Il18r1 Jak2 Klrg1 Ly86 Ly96 Pglyrp1 Samhd1 Serpina3g Serpina3n Serping1 Sp110 Timp3	23(396)	285(37681)	6.06E-14	0.2013	GO:0045087 innate immune response (BP)<br>GO:0006958 complement activation, classical pathway (BP)<br>GO:0006954 inflammatory response (BP)<br>GO:0010951 negative regulation of endopeptidase activity (BP)<br>GO:0010466 negative regulation of peptidase activity (BP)
Metagroup 10	Adipoq Aif1 Aoah Aoc3 Apoe Car3 Ccl2 Cd24a Clec5a Clec9a Cxcl16	34(396)	694(37681)	1.52E-13	-0.0038	GO:0032496 response to lipopolysaccharide (BP)<br>GO:0043407 negative regulation of MAP kinase activity (BP)

	Dnm1 Enpp2 Fndc1 Ifng Igf1 Il12rb2 Il2ra Irak3 Irg1 Jak2 Ly96 Mapkapk3 Mgst1 Nckap1l Peli1 Pik3r1 Ptprj Rgs2 Sod2 Stk17b Stk3 Tnfaip3 Zfp36					GO:0050728 negative regulation of inflammatory response (BP)<br>GO:0048662 negative regulation of smooth muscle cell proliferation (BP)<br>GO:0001934 positive regulation of protein amino acid phosphorylation (BP)<br>GO:0006898 receptor-mediated endocytosis (BP)<br>GO:0006916 anti-apoptosis (BP)<br>GO:0007243 protein kinase cascade (BP)<br>GO:0006979 response to oxidative stress (BP)<br>GO:0006950 response to stress (BP)
Metagroup 11	Fgl2 Gzma Gzmb Gzmc Gzme Gzmf Gzmg Lyz1 Lyz2	9(396)	22(37681)	6.30E-13	0.8095	GO:0019835 cytolysis (BP)
Metagroup 12	Abca1 C3 Cd48 Clec7a Elmo1 Fcer1g Fcgr2b Fcgr3 Fyb Lcp2 Ptx3	11(396)	43(37681)	6.40E-13	0.4737	GO:0045576 mast cell activation (BP)<br>GO:0006911 phagocytosis, engulfment (BP)<br>GO:0050766 positive regulation of phagocytosis (BP)
Metagroup 13	Cd28 Cd38 Cd3d Cd3e Cd4 Cd74 Ctla4 Cxcl12 Cybb Dock2 Fas Ikzf1 Il7r Malt1 Mgst1 Nckap1l Peli1 Pf4 Ptprc Sod2	20(396)	281(37681)	2.67E-11	0.0841	GO:0045060 negative thymic T cell selection (BP)<br>GO:0045059 positive thymic T cell selection (BP)<br>GO:0042098 T cell proliferation (BP)<br>GO:0006955 immune response (BP)<br>GO:0030890 positive regulation of B cell proliferation (BP)<br>GO:0050853 B cell receptor signaling pathway (BP)<br>GO:0030217 T cell differentiation (BP)<br>GO:0006461 protein complex assembly (BP)<br>GO:0042493 response to drug (BP)
Metagroup 14	Adipoq Camk4 Cblb Ccr2 Ccrl2 Cd28 Cd74 Cxcr2 Fas Lat P2ry14 Sh2d1a Stap1	13(396)	111(37681)	1.86E-10	0.2312	GO:0006955 immune response (BP)<br>GO:0006968 cellular defense response (BP)<br>GO:0009967 positive regulation of signal transduction (BP)<br>GO:0006954 inflammatory response (BP)
Metagroup 15	Anpep Apoe Arhgap15 Ccl2 Ccl3 Ccl5 Ccr2 Cd36 Cd74 Cyp1b1 Dock2 Fgd4 Gbp4 Gbp5 Gbp8 H2-Ab1 Hif1a Il1a Ly96 Pf4 Pstpip2 Thbs2	22(396)	432(37681)	1.63E-09	0.1302	GO:0006955 immune response (BP)<br>GO:0006954 inflammatory response (BP)<br>GO:0006874 cellular calcium ion homeostasis (BP)<br>GO:0019221 cytokine-mediated signaling pathway (BP)<br>GO:0071346 cellular response to interferon-gamma (BP)<br>GO:0031663 lipopolysaccharide-mediated signaling pathway (BP)<br>GO:0016525 negative regulation of angiogenesis (BP)<br>GO:0007010 cytoskeleton organization (BP)<br>GO:0008360 regulation of cell shape (BP)<br>GO:0001525 angiogenesis (BP)
Metagroup 16	Adam23 Ccl5 Cd2 Cd24a Clec7a Cyfip2 Fcer1g Itga1 Itga4 Itgax Itgb2 Lat Plek Ptprc	14(396)	160(37681)	1.83E-09	0.4028	GO:0007229 integrin-mediated signaling pathway (BP)<br>GO:0007159 leukocyte adhesion (BP)<br>GO:0016337 cell-cell adhesion (BP)
Metagroup 17	Adipoq C3 Ccl3 Ccl5 Ccr3 Cd36 Cd74 Clec4e Clec4n Clec5a Clec9a Fcer1g Il1a Il33 Itgb2 Malt1 Peli1	17(396)	257(37681)	2.54E-09	0.1289	GO:0050715 positive regulation of cytokine secretion (BP)<br>GO:0032755 positive regulation of interleukin-6 production (BP)<br>GO:0043123 positive regulation of I-kappaB kinase/NF-kappaB cascade (BP)<br>GO:0045766 positive regulation of angiogenesis (BP)<br>GO:0070374 positive regulation of ERK1 and ERK2 cascade (BP)
Metagroup 18	2610018G03Rik Aif1 Ccl5 Cd24a Cd38 Cd3g Dpp4 Dusp22 Fas Hif1a Hoxd13 Igf1 Ikzf3 Irak3 Itgb2 Malt1 Prkcq Prlr Ptprc Socs2 Tnfrsf9 Xdh	22(396)	469(37681)	7.28E-09	0.0401	GO:0007595 lactation (BP)<br>GO:0042981 regulation of apoptosis (BP)<br>GO:0042127 regulation of cell proliferation (BP)<br>GO:0048661 positive regulation of smooth muscle cell proliferation (BP)<br>GO:0051092 positive regulation of NF-kappaB transcription factor activity (BP)<br>GO:0001666 response to hypoxia (BP)
Metagroup 19	C3 F10 F13a1 F5 F7 Igf1 Papss2 Ptprj Serping1 Stk3	10(396)	105(37681)	1.74E-07	0.5021	GO:0007596 blood coagulation (BP)<br>GO:0051897 positive regulation of protein kinase B signaling cascade (BP)

Supplementary Table 2. Gene ontology biological process terms enriched in the immune module. The analysis was performed with GeneTermLinker software (http://gtlinker.cnb.csic.es/).

	Genes	#list	#ref_list	adjusted pValue	Silhouette Width	Terms
Metagroup 1	Anapc13 Bub1 Bub1b Ccna2 Ccnb2 Ccnd1 Cdc25c Cdk1 Cdk6 Chek1 Dbf4 Ei24 Hdac2 Mad2l1 Mcm4 Mcm6 Orc2 Orc6 Rbl1 Tfdp1 Ttk	21(507)	166(37681)	1.22E-14	0.2943	04110 Cell cycle<br>04115 p53 signaling pathway
Metagroup 2	Aaas Dkc1 Eif1ax Eif3b Eif3c Gnl3 Nop58 Nup107 Nup155 Nup160 Nup205 Nup43 Ran Rexo2 Riok2 Rnps1 Rpp30 Wdr43 Xpo1	19(507)	214(37681)	1.23E-10	0.2774	03013 RNA transport<br>03008 Ribosome biogenesis in eukaryotes
Metagroup 3	Actn1 Akt3 Birc3 Brca2 Cav1 Cav2 Ccnd1 Cdk6 Cks1b Dock1 Fgf14 Fgfr2 Flnb Gli3 Grlf1 Hdac2 Itgav Lama4 Lamb1 Ldlr Mecom Met Mmp9 Nos2 Parva Prkca Vcl	27(507)	508(37681)	2.02E-09	0.2042	04510 Focal adhesion<br>05200 Pathways in cancer<br>05222 Small cell lung cancer<br>05145 Toxoplasmosis
Metagroup 4	Adcy8 Akt3 Anapc13 Aurka Bub1 Ccna2 Ccnb2 Cdc25c Cdk1 Fbxo5 Mad2l1	11(507)	143(37681)	4.11E-06	0.7246	04110 Cell cycle<br>04914 Progesterone-mediated oocyte maturation<br>04114 Oocyte meiosis
Metagroup 5	Acaca Actn1 Akt3 Cttn Epb4.1l2 Epb4.1l3 Ereg Fasn Fgf14 Fgfr2 Hbegf Irs1 Jam2 Mecom Ppp2r2d Prkar2b Prkca	17(507)	352(37681)	7.11E-06	-0.003	04530 Tight junction<br>04010 MAPK signaling pathway<br>05200 Pathways in cancer<br>04012 ErbB signaling pathway<br>04910 Insulin signaling pathway
Metagroup 6	Adcy8 Ak5 Aprt Dctd Gmps Mcm4 Mcm6 Papss1 Pnpt1 Pola1 Pole Polr3f Rrm1	13(507)	213(37681)	7.28E-06	0.4132	00230 Purine metabolism<br>03030 DNA replication<br>00240 Pyrimidine metabolism
Metagroup 7	Actn1 Diap3 Dock1 F2r Fgf14 Fgfr2 Grlf1 Itgav Jam2 Met Mmp9 Prkca Ptprm Thy1 Vcl	15(507)	330(37681)	4.87E-05	0.1726	04670 Leukocyte transendothelial migration<br>04810 Regulation of actin cytoskeleton<br>04510 Focal adhesion<br>04520 Adherens junction
Metagroup 8	Arg1 Cyp2b10 Cyp2e1 Fmo1 Gsto1 Idh2 Maoa Nos2 Odc1 Rrm1	10(507)	163(37681)	7.83E-05	0.1334	00480 Glutathione metabolism<br>00330 Arginine and proline metabolism<br>00982 Drug metabolism - cytochrome P450
Metagroup 9	Akt3 Brca2 Ccnd1 Cdk6 Fgf14 Hdac2 Mecom Met Prkca	9(507)	133(37681)	8.39E-05	0.4894	05200 Pathways in cancer<br>05220 Chronic myeloid leukemia<br>05214 Glioma<br>05223 Non-small cell lung cancer<br>05218 Melanoma<br>05212 Pancreatic cancer
Metagroup 10	Actn1 Arg1 Lama4 Lamb1 Nos2 Prkca Serpinb6b Vcl	8(507)	114(37681)	0.000160926	0.4672	05146 Amoebiasis<br>04510 Focal adhesion<br>05200 Pathways in cancer
Metagroup 11	Abca8a Abca9 Abcb1b Abcd2 Adcy8 Ldlr Slc4a4	7(507)	105(37681)	0.000560655	0.2159	02010 ABC transporters<br>04976 Bile secretion
Metagroup 12	Asap1 Cav1 Cav2 Cttn Dock1 F2r Fgfr2 Ldlr Met Tfrc Vcl	11(507)	265(37681)	0.00101716	0.5042	04510 Focal adhesion<br>05100 Bacterial invasion of epithelial cells<br>04144 Endocytosis
Metagroup 13	Cd34 Cdh2 Itgav Jam2 Lama4 Lamb1 Ptprm Sdc2 Vcan	9(507)	217(37681)	0.00286119	0.1041	04514 Cell adhesion molecules (CAMs)<br>04512 ECM-receptor interaction

Supplementary Table 3. Biological pathways (KEGG) enriched in the cancer module. The analysis was performed using GeneTermLinker software (http://gtlinker.cnb.csic.es/).

cMap name and cell line	mean	n	enrichment	p	specificity
harmine - MCF7	0.945	2	0.988	0.00026	0.0066
galantamine - MCF7	0.911	2	0.972	0.00125	0.0168
tomatidine - MCF7	0.89	2	0.962	0.00256	0.0164
pyridoxine - MCF7	0.882	2	0.985	0.00044	0.0073
astemizole - PC3	0.869	2	0.972	0.00123	0.0337
protoveratrine A - MCF7	0.868	2	0.975	0.00099	0
alexidine - PC3	0.857	2	0.967	0.00185	0.033
meticrane - PC3	0.855	2	0.958	0.00308	0.0303
resveratrol - MCF7	0.811	6	0.805	0.00008	0.0895
phenoxybenzamine - MCF7	0.808	3	0.929	0.00064	0.1921
thioridazine - PC3	0.719	5	0.718	0.00409	0.1636
0175029-0000 - PC3	0.705	4	0.81	0.00247	0.0764
alpha-estradiol - MCF7	0.67	9	0.624	0.00064	0.042
vorinostat - MCF7	0.669	7	0.738	0.00022	0.3158
prochlorperazine - MCF7	0.661	9	0.572	0.00252	0.1841
trichostatin A - PC3	0.654	55	0.529	0	0.44
tretinoin - MCF7	0.602	13	0.578	0.00008	0.0659
trichostatin A - MCF7	0.523	92	0.382	0	0.7156
trifluoperazine - MCF7	0.491	9	0.599	0.00116	0.1971
LY-294002 - MCF7	0.487	34	0.367	0.0002	0.3421
sirolimus - MCF7	0.396	25	0.401	0.0004	0.2711

Supplementary Table 4. Identification of drug repurposing candidates that can promote treatment response using the cMap build 02 database. The database was queried with a gene signature derived from the immune and cancer modules. Mean represents the arithmetic mean of the connectivity scores, which are defined as the strength of the overlap between the perturbation signature in cMap with the gene expression signature in the immune/cancer modules, over the set of experiments for that drug and cell line (n). The enrichment is a measure of the enrichment of those experiments amongst a ranked list of all experiments. P is a permutation p-value for the enrichment. Specificity is a measure of the uniqueness of the connectivity in comparison to experimentally-derived signatures from MSigDB (high values indicate low specificity).

Upstream Regulator	Molecule Type	Predicted Activation State	Activation z-score	Bias Term	Bias-corrected z-score	p-value of overlap	Target molecules in dataset
tretinoin	chemical - endogenous mammalian	Activated	3.808	-0.021	4.020	1.24E-24	ABCA1,ABI3BP,ACACA,ACP5,ACSL1,AFF2,ANPEP,ANXA5,APOE,ARG1,BIRC3,BTG2,C3,Ccl2,CCL22,CCNA2,CCND1,CCRL2,CCT5,CD34,CD36,CD38,CD53,CD74,CD86,CDK6,CDKN3,Chil3/Chil4,CHL1,CP,CPE,CRABP1,CREG1,CSE1L,CTLA4,CYBB,CYP2B6,DLGAP5,DPP4,DPYSL3,DTL,EIF3B,EIF3C/EIF3CL,ENPP2,ERCC1,ERRFI1,FABP4,FCER1G,FOSL1,GAP43,GAS2,GFM1,GIMAP6,GM2A,GNAO1,GPRC5A,GZMA,GZMB,HBEGF,HMOX1,HOXD13,HSD11B1,IFITM1,IFNG,IFNGR1,IGF1,IGFBP7,IGHM,IGJ,IKZF1,IL1A,INHBA,IRS1,ISLR,ITGA1,ITGA4,ITGAX,ITGB2,JAK2,KIF11,KIF23,LAMA4,LAMB1,LIPA,MAPK6,MARCKS,MECOM,MEIS2,MGP,MMP13,MMP3,MMP9,MPP6,MSLN,MYBL2,NAE1,NOS2,NR2F1,NR2F2,NRP1,ODC1,PAM,PDCD4,PENK,PIK3CG,PLA2G7,PLEK,PLIN2,PLK2,POSTN,PRKCA,PTX3,RAN,RBL1,RBL2,REL,S100A9,SAMHD1,SELL,SERPING1,SLAMF7,SLC12A2,SLC7A2,SLC9B2,SP110,SRGN,STAT4,STRA6,TF,TFRC,TNFAIP3,TOP2A,TUBB3,TUBGCP2,UBE2C,VCL,XRCC5
phorbol myristate acetate	chemical drug	Activated	3.391	-0.027	3.652	1.11E-22	ABCA1,ADAMTS1,APRT,AURKA,AURKB,BIRC3,BLM,BTG2,C3,CAV1,CAV2,Ccl2,CCL3L1/CCL3L3,CCL5,CCNA2,CCND1,CCR2,CCR3,CD28,CD36,CD4,Cd55/Daf2,CDK1,CPE,CTLA4,CTTN,Cxcl11,CXCL3,CXCR2,CYBB,CYP2E1,DTL,E330013P04Rik,EFNB2,EGR2,ERCC1,ERRFI1,FAS,FCER1G,FOSL1,GAP43,GON4L,GZMH,HBEGF,HIF1A,HLADRA,HMGA2,HSD11B1,ICOS,IFNG,IFNGR1,IGF1,IL12RB2,IL1A,IL2RA,INHBA,IRAK3,ITGAX,ITGB2,ITM2A,LAT,LDLR,LIPA,LPL,LYVE1,Lyz1/Lyz2,MME,MMP12,MMP13,MMP3,MMP9,NF1,NFIL3,NOS2,NRP1,ODC1,PECAM1,PF4,PGAP1,PMCH,PPBP,PRKAR2B,PRKCA,PRKCQ,PRKD1,PTPRJ,RANBP1,RASGRP1,REL,RGS1,RGS2,S100A9,SELL,SERPINA1,SERPINA3,SLC12A2,SLC7A11,SOD2,SPIC,SRGN,TFDP1,THY1,TNFAIP3,VCAN,VCL,ZFP36
AGN194204	chemical drug	Activated	3.555	-0.053	3.807	4.66E-21	AURKA,CCL3L1/CCL3L3,CCNA2,Cd24a,CD3E,CDK1,CYP51A1,CYP7B1,DGKA,EMP1,FDFT1,FDPS,FNBP1,GNAI1,Gp49a/Lilrb4,GPR65,GZMB,ICOS,IGHM,IL2RA,IL2RB,IL7R,ITGB2,ITM2A,KIF20A,KIF2C,KIF4A,LIFR,LPL,MAD2L1,MKI67,MMP9,PDCD4,PENK,PLIN2,PRC1,SCIN,SMC2,STRA6,TOP2A
bleomycin	chemical drug	Activated	3.651	-0.056	3.958	3.83E-15	BLM,CCL17,Ccl2,CCL22,CCL3L1/CCL3L3,CCL5,Ccl8,Ccl9,CCR2,CD86,CTSE,CTSS,CXCL1,F2R,FABP4,FAP,FAS,FOSL1,HBEGF,IFNG,IGF1,IL1A,ITGA1,MMP12,MMP9,Retnla,Saa3,SOCS2,STK17B,TIMP3,UBE2C,UHRF1
calcitriol	chemical drug	Activated	3.797	0.006	3.758	5.81E-15	AAAS,ANLN,BUB1B,C3,CCNA2,CCND1,CDCA8,CDK1,CLEC2D,CSE1L,CXCL3,CYP2B6,ECT2,ERRFI1,FABP4,FAS,FASN,FIGNL1,FOXM1,HBEGF,IFITM1,IFNG,IGF1,IL1A,IL1RN,IL2RA,ITGA4,ITGAX,KIF20A,KIF23,LPL,LTBP1,MAD2L1,MCM4,MELK,MMS22L,NCAPD2,NDC1,NUP43,ODC1,PBK,PDCD4,PMEPA1,POLE,PRC1,PRKCA,RACGAP1,REL,S100A9,SERPINA1,SMC2,SUV39H1,TNFAIP3,TPX2,VCL,XPO1

Supplementary Table 5. Prior knowledge based identification of drugs that are predicted to promote treatment response. Upstream regulator analysis (Ingenuity Systems) was employed to identify drugs that based on experimental findings reported in the literature are known to regulate expression levels of genes in the immune/cancer modules. The overlap p-value is based on enrichment of genes whose expression levels are known to be modulated by a given drug. The activation Z-score is a measure of the pattern match between the direction of the gene expression changes (up/down regulation) and the predicable pattern from prior knowledge.

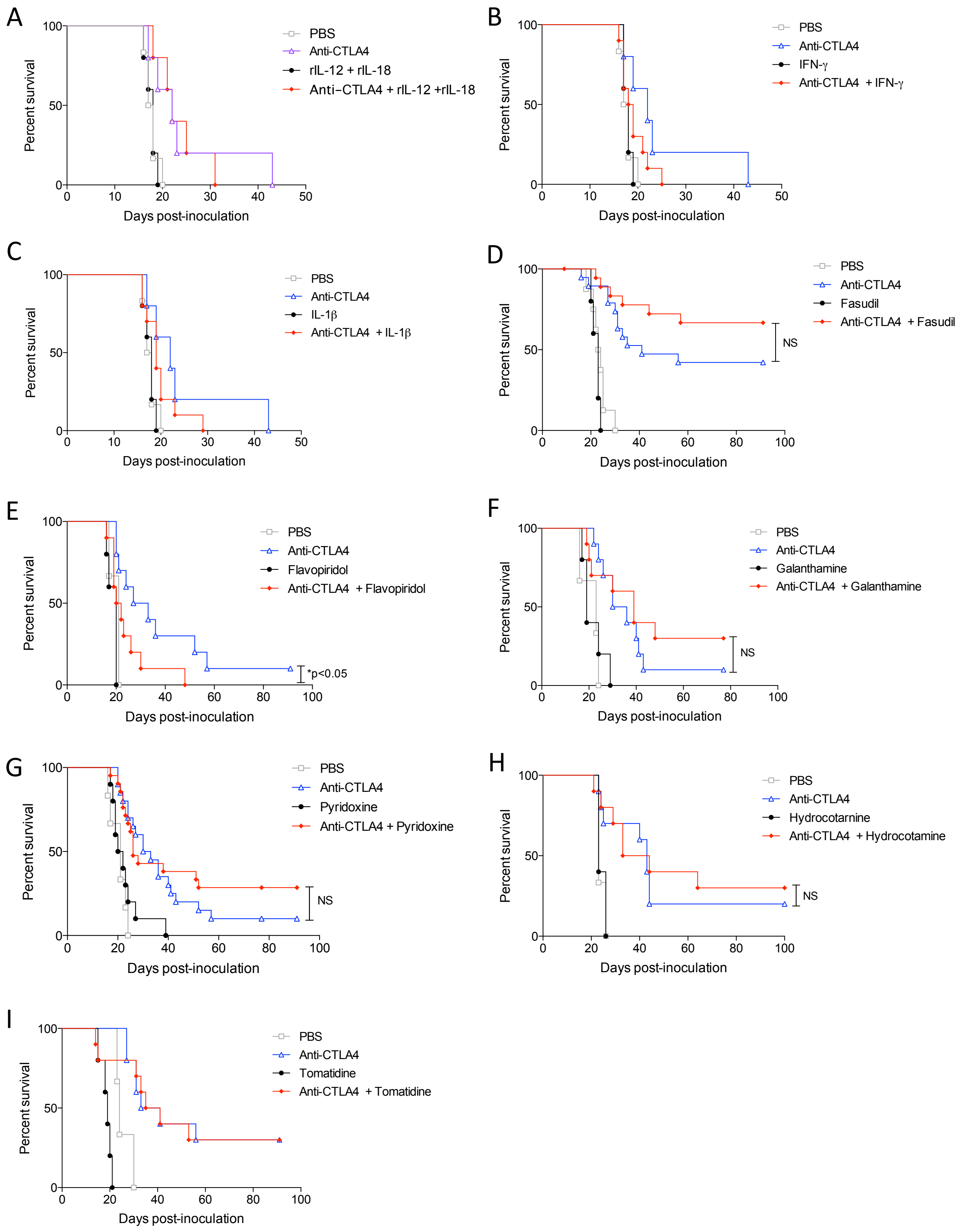

Supplementary Fig. 2. Targeting response-associated hubs and modules aimed at improving the therapeutic efficacy of CTLA4 blockade. AB1-HA bearing mice were

treated with anti-CTLA4 (100 µg on day 10) in combination with therapeutics targeted at specific response-associated hubs (**a-e**, also see Fig. 3 in the article) or drugs based on overlap with drug-perturbation signatures in the cMap database[1] (**f-i**, also see Fig. 4 in the article). (**a-c**) Recombinant murine cytokines IL-12, IL-18, IFNγ and IL-1β were used, since they were all hubs within the immune module and upregulated in responders (a representative of two independent experiments with n = 5-10 mice per arm is shown; for clarity the experiment is divided over three separate graphs). No significant differences were observed. (**d**) We observed that several hub genes in the cancer module were involved in Rho kinase activation, and were downregulated in responders only. For this reason, we treated mice with anti-CTLA4 in combination with fasudil, a Rho kinase-specific inhibitor that is used in patients with cerebral vasospasm[2] (two separate experiments are combined in this graph, n = 20 mice per arm for anti-CTLA4-containing regimes, n = 8 for control arms). Fasudil alone did not result in any growth delay, but in combination with anti-CTLA4 it increased the response rate consistently in several independent experiments, however without reaching statistical significance. This is partly explained by the overlapping curves in the beginning of the curve, as a result of increasing the response rates without affecting survival in all mice[3]. Since the difference in survival did not reach statistical significance after treating 20 mice per arm, we did not continue testing this drug. (**e**) CDKN1 inhibitor flavopiridol was tested in combination with anti-CTLA4 since CDKN1 was a hub within the cancer module, which was downregulated in responders. The outcome after combination treatment was significantly worse than after anti-CTLA4 alone. This can be explained by negative effects on lymphocyte responses, and thus the immune model, as has been reported before by others[4]. This formed an important reason for us to investigate whether we could use pleiotropic drugs that would target the immune and cancer module at the same time, based on an overlap in drug-perturbation signatures in the cMap database and the gene expression in our dataset of genes with in the immune and cancer module, as described in the article. We identified several drug candidates in this manner (see also Fig. 4 in the article), including (**f**) Alzheimer's drug Galantamine (a representative of two independent experiments, n = 10 mice for anti-CTLA4-containing regimes, n = 5 for control arms), (**g**) vitamine B/Pyridoxine (two separate experiments are combined in this graph, n = 20 mice per arm for anti-CTLA4-containing regimes, n = 13 for pyridoxine and 6 for PBS arms), (**h**) analgesic Hydrocotamine (a representative of two independent experiments, n = 10 mice for anti-CTLA4-containing regimes, n = 5 for control arms) and (**i**) plant alkaloid Tomatidine (one experiments, n = 10 mice for anti-CTLA4-containing regimes, n = 5 for control arms). Although again for some of these drugs we indeed consistently observed over multiple experiments that response rates were increased, this did not translate in a significant difference in overall survival.

Supplementary References

1. Lamb, J. et al. The Connectivity Map: using gene-expression signatures to connect small molecules, genes, and disease. *Science* **313**, 1929-1935 (2006).

2. Shi, J. & Wei, L. Rho kinases in cardiovascular physiology and pathophysiology: the effect of fasudil. *Journal of cardiovascular pharmacology* **62**, 341-354 (2013).

3. Wolchok, J.D. et al. Development of ipilimumab: a novel immunotherapeutic approach for the treatment of advanced melanoma. *Annals of the New York Academy of Sciences* **1291**, 1-13 (2013).

4. Arguello, F. et al. Flavopiridol induces apoptosis of normal lymphoid cells, causes immunosuppression, and has potent antitumor activity In vivo against human leukemia and lymphoma xenografts. *Blood* **91**, 2482-2490 (1998).
